# Atomic structures and functional implications of the archaeal RecQ-like helicase Hjm

**DOI:** 10.1186/1472-6807-9-2

**Published:** 2009-01-22

**Authors:** Takuji Oyama, Hayato Oka, Kouta Mayanagi, Tsuyoshi Shirai, Kyoko Matoba, Ryosuke Fujikane, Yoshizumi Ishino, Kosuke Morikawa

**Affiliations:** 1 The Takara Bio Endowed Division, Institute for Protein Research, Osaka University, Open Laboratories of Advanced Bioscience and Biotechnology (OLABB), 6-2-3 Furuedai, Suita, Osaka 565-0874, Japan; 2 Department of Genetic Resources Technology, Faculty of Agriculture, Kyushu University, 6-10-1 Hakozaki, Fukuoka-shi, Fukuoka, 812-8581, Japan; 3 Division of Structural Biology, Medical Institute of Bioregulation, Kyushu University, Maidashi 3-1-1, Higashi-ku, Fukuoka 812-8582, Japan; 4 Department of Bioscience, Nagahama Institute of Bioscience and Technology, 1266 Tamura, Nagahama 526-0829, Japan; 5 Laboratory of Protein Synthesis and Expression, Institute for Protein Research, 3-2 Yamadaoka, Suita, Osaka 565-0874, Japan; 6 BIRD, JST, Japan; 7 Research & Develop center, Terumo Corporation, 1500, Inokuchi, Nakai-machi, Ashigarakami-gun, Kanagawa, 259-0151, Japan; 8 Univ. Paris-Sud, Institut de Génétique et Microbiologie, CNRS, UMR 8621, F-91405 Orsay Cedex, France

## Abstract

**Background:**

*Pyrococcus furiosus *Hjm (*Pfu*Hjm) is a structure-specific DNA helicase that was originally identified by *in vitro *screening for Holliday junction migration activity. It belongs to helicase superfamily 2, and shares homology with the human DNA polymerase Θ (PolΘ), HEL308, and *Drosophila *Mus308 proteins, which are involved in DNA repair. Previous biochemical and genetic analyses revealed that *Pfu*Hjm preferentially binds to fork-related Y-structured DNAs and unwinds their double-stranded regions, suggesting that this helicase is a functional counterpart of the bacterial RecQ helicase, which is essential for genome maintenance. Elucidation of the DNA unwinding and translocation mechanisms by *Pfu*Hjm will require its three-dimensional structure at atomic resolution.

**Results:**

We determined the crystal structures of *Pfu*Hjm, in two apo-states and two nucleotide bound forms, at resolutions of 2.0–2.7 Å. The overall structures and the local conformations around the nucleotide binding sites are almost the same, including the side-chain conformations, irrespective of the nucleotide-binding states. The architecture of Hjm was similar to that of *Archaeoglobus fulgidus *Hel308 complexed with DNA. An Hjm-DNA complex model, constructed by fitting the five domains of Hjm onto the corresponding Hel308 domains, indicated that the interaction of Hjm with DNA is similar to that of Hel308. Notably, sulphate ions bound to Hjm lie on the putative DNA binding surfaces. Electron microscopic analysis of an Hjm-DNA complex revealed substantial flexibility of the double stranded region of DNA, presumably due to particularly weak protein-DNA interactions. Our present structures allowed reasonable homology model building of the helicase region of human PolΘ, indicating the strong conformational conservation between archaea and eukarya.

**Conclusion:**

The detailed comparison between our DNA-free *Pfu*Hjm structure and the structure of Hel308 complexed with DNA suggests similar DNA unwinding and translocation mechanisms, which could be generalized to all of the members in the same family. Structural comparison also implied a minor rearrangement of the five domains during DNA unwinding reaction. The unexpected small contact between the DNA duplex region and the enzyme appears to be advantageous for processive helicase activity.

## Background

DNA helicases are enzymes that translocate along DNA and unwind double-stranded regions in an ATP-dependent manner [1,2]. They play crucial and universal roles in DNA metabolism, such as DNA replication and recombinational repair. As a consequence of their physiologically important functions, many reports have been published regarding protein characterization and catalytic mechanisms, including the relationships between enzymatic dysfunctions and several human genetic diseases [3,4]. Our on-going structural analysis of the late stage of homologous recombination, such as the RuvABC-Holliday junction (HJ) complex [5], tempted us to investigate the molecular machinery involved in Holliday junction processing in eukaryotes. We also noticed that the archaeal proteins involved in DNA metabolism generally have amino acid sequences and three-dimensional (3D) structures that are highly similar to their eukaryotic homolog. The proteins from the hyperthermophilic archaea, including *Pyrococcus furiosus*, are more advantageous for structural studies than their eukaryotic counterparts, because of their remarkable thermal stability. In fact, we were the first group to successfully identify the Holliday junction resolvase from archaea, which we designated as Hjc [6], and we also determined its crystal structure by X-ray analysis [7]. A subsequent screening study for a new protein factor that stimulates the HJ resolving activity by Hjc led to the identification of a new protein factor, termed Hef [8]. Biochemical and sequence analyses revealed that this protein should be classified as an XPF/Rad1/Mus81 nuclease, which bears endonuclease activity specific for flap or fork structures. Interestingly, the full-length Hef molecule contains a Super family 2 (SF2) helicase at the amino terminus. We determined the crystal structures of each region that individually folds into a distinct, rigid architecture, such as the helicase region, the nuclease domain, and the C-terminal domain containing the two repeated HhH motifs [9-11]. The combined approach of structural and functional analyses of the nuclease regions also revealed the bipartite substrate recognition mode, which is quite likely to be conserved in the XPF/Rad1/Mus81 nuclease family. Intriguingly, the human Hef ortholog was found to be an important component of the FANC core complex, which plays a crucial role in the Fanconi Anemia-related DNA repair process responding to cross-link damage [12-14].

In parallel with these studies, we initiated experiments to identify the branch migration activity of the Holliday junction in archaea. In *P. furiosus*, we successfully indentified a novel DNA helicase, which we designated as Hjm (pf0677), according to its functional activity, Holliday junction migration [15]. Its primary structure of 720 amino acids indicated that the Hjm helicase belongs to SF2, and was intriguingly found to share significant similarity to the helicase-like regions of the human DNA polymerase Θ (PolΘ), HEL308, and Drosophila Mus308 proteins, which are all involved in DNA repair. Hjm appears to be unique to archaea, because of the lack of sequence similarity to proteins from bacteria and yeast. However, it was recently found that this structure-specific helicase preferentially binds to fork-related Y-structured DNAs and unwinds their double-stranded regions. Additionally, Hjm partially complements the RecQ function in *E. coli dnaE486recQ *mutant cells *in vivo *[16]. Similar results were also reported for another archaeal homologous helicase from *Methanothermobacter thermautotrophicus *[17]. These results suggest that Hjm may be a functional counterpart of the RecQ helicases in archaea. The functional interaction of Hjm with PCNA also revealed that this helicase could participate in a reconstituted replisome to restart a stalled replication fork [16]. Most recently, the crystal structure of the archaeal homolog of Hel308, from *Archaeoglobus fulgidus*, was determined in both the DNA-free and DNA-complexed states [18]. Another structure of Hel308 from *Sulfolobus solfataricus *was also reported, and a unique role for the small C-terminal domain to regulate its unwinding activity was proposed in combination with biochemical studies [19]. Despite these intriguing findings, many aspects of the Hjm helicase, such as its actual substrates *in vivo *and its ATP-dependent unwinding mechanism of DNA duplexes, still remain elusive.

In order to obtain more detailed and clearer insights into the 3D structure and the helicase action at the atomic level, we determined the crystal structure of *Pfu*Hjm, in two apo-states at 2.0 and 2.4 Å resolution, in the ADP-bound form at 2.4 Å, and in the ATP-analog bound form at 2.7 Å. In combination with single particle electron microscopy of the enzyme complexed with a putative synthetic DNA substrate, the atomic structure revealed clearer views of the functional and structural aspects of each domain, such as DNA substrate recognition and nucleotide binding, in comparison with the structural data of the previously reported Hel308 helicases.

## Results and discussion

### Overview of the structure

We obtained the two different *Pfu*Hjm crystals (Forms 1 and 2) in the nucleotide free-state, and determined their structures at 2.4 Å (Form 1) and 2.0 Å (Form 2) resolutions, respectively (Table 1). In the Form 1 crystal, the C-terminal 60 residues, about two thirds of the C-terminal domain, are missing in the final model, presumably because of structural disorder. On the other hand, in the Form 2, we could build the model of almost the entire molecule except for the C-terminal twenty residues. Although we could not obtain cocrystals with nucleotides, we soaked ATP analogs into the crystals, and successfully determined the nucleotide complex structures. The structures of the two apo-forms are quite similar, with a root-mean square deviation (rmsd) of 1.05 Å for the corresponding 651 C_α _atoms. Nucleotide binding to the protein also causes no large structural change; The overall rmsd value between the apo- and ATPγS-soakd states is 0.30 Å in Form 1, and similarly that between the apo- and AMPPCP-bound structures is 0.40 Å in Form 2.

**Table 1:** Data collection, phasing, and refinement

***Data Collection Summary***							
	Form 1 Native Apo	ATPγS soaked form	Derivative Ta_6_Br_14_	SeMet	K_2_PtCl_4_	Form 2 Apo	AMPPCP soaked form

Wavelength (Å)	1.0000	1.0000	1.2553	0.9792	1.0000	1.0000	1.0000
Resolution (Å)	50.0-2.40	50.0-2.40	50.0-3.00	50.0-2.50	50.0-2.60	50.0-2.00	50.0-2.7
(Highest shell)	(2.49-2.40)	(2.49-2.40)	(3.11-3.00)	(2.59-2.50)	(2.69-2.60)	(2.07-2.00)	(2.80-2.70)
Measured reflections	123829	67944	58990	101649	65378	195368	84923 (8506)
Unique reflections	31237 (3096)	29610 (3006)	16100 (1600)	27042 (2770)	24345 (2421)	51591 (4787)	22378 (2240)
Completeness	99.8 (99.7)	96.2 (98.3)	99.9 (99.9)	97.1 (100.0)	97.9 (98.2)	97.6 (91.1)	98.8 (100.0)
*I*/σ(*I*)	15.1 (7.7)	14.9 (5.3)	21.9 (6.4)	12.8 (5.8)	14.1 (5.3)	9.4 (1.8)	16.5 (6.9)
Redundancy	3.9 (3.9)	2.3 (2.2)	3.7 (3.7)	3.8 (3.8)	2.7 (2.6)	3.8 (3.2)	3.8 (3.8)
*R*_merge_	7.1 (39.1)	7.0 (44.5)	5.3 (14.1)	7.8 (43.6)	7.4 (45.4)	7.0 (45.0)	12.0 (40.2)

***MIRAS Phasing Statistics***							

*R*_iso_(*F*) (%)			11.1	18.6	20.6		
Number of Sites			8	1	5		
Resolution (Å)			50.0-3.9	50.0-3.9	50.0-3.9		
Phasing Power (Centric/Acentric)			0.814/0.723	1.799/1.894	1.036/0.859		
Figure of merit (Centric/Acen.)	0.74/0.64						

***Refinement***							
Resolution (Å)	50.0-2.40	5.0-2.40				50.0-2.00	50.0-2.70
*R*_work_/*R*_free _^a ^(%)	22.8/29.9	21.6/29.0				22.3/25.8	23.1/29.4
Number of atoms							
Protein	5202	5374				5603	5599
Water	90	113				300	65
Ligand	25	47				-	31
Average B-factor (Å^2^)							
Protein	41.3	38.9				27.8	37.5
Water	42.2	40.2				32.1	32.1
Ligand	60.9	58				-	71.2
r.m.s.d.							
Bond Lengths(Å)	0.008	0.009				0.007	0.008
Angles(°)	1.3	1.3				1.3	1.3
PDB code	2ZJ2	2ZJ5				2ZJ8	2ZJA

^a^*R*_free _was calculated using 5% of the total reflections, which were chosen randomly and omitted from the refinement.

**Figure 1:**
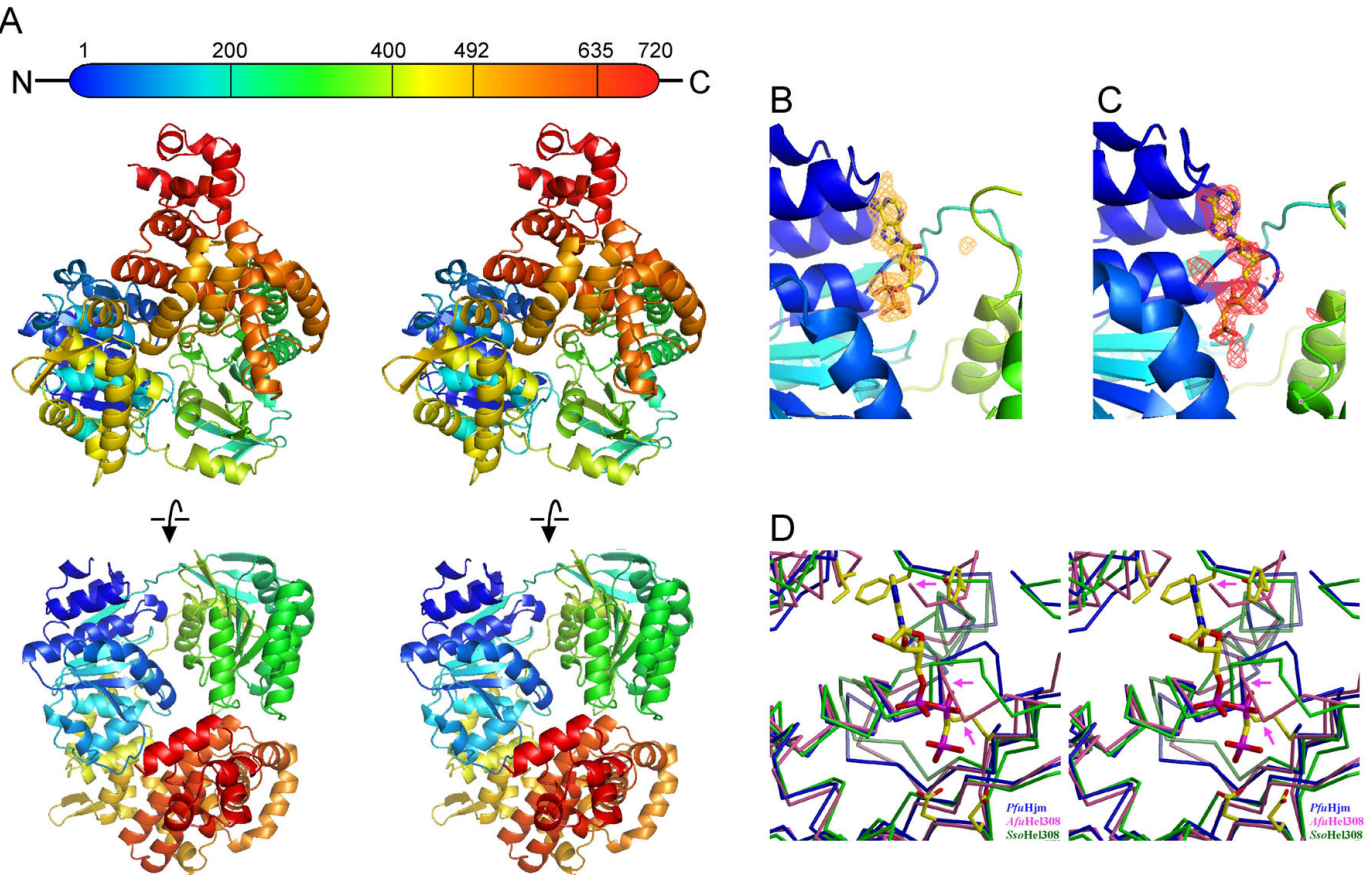
**Structure of *Pyrococcus furiosus *Hjm**. (A) The Form 2 apo structure is shown as a ribbon representation with a color spectrum from blue (N-terminus) to red (C-terminus). (B) and (C) Omit |*F*_o_-*F*_c_| maps (2.5 σ) for ADP in Form 1 (B) and AMPPCP in Form 2 (C) are shown with the final models. (D) Comparison of the nucleotide binding sites between Hjm and Hel308. Hjm is represented in blue, and the key residues interacting with the ATP analog are highlighted. Segments of *A. fulgidus *Hel308 (pink) should undergo structural changes to bind the nucleotide, while *S. solfataricus *Hel308 (green) could bind the nucleotide with slight rearrangements in the pocket. *A. fulgidus *Hel308 residues, which should sterically clash with the nucleotide, are indicated by magenta arrows.

*Pfu*Hjm folds into five domains (domains 1 to 5) with dimensions of approximately 70 × 50 × 30 Å. The protein possesses a concave surface on the front-view side and a hole (about 10 Å diameter) at the center of the molecule (Figure 1a). The two N-terminal domains 1 (residues 1–197) and 2 (residues 198–399) form typical helicase domains with a cleft between them, as commonly observed in the helicase superfamily. The seven conserved helicase sequence motifs [20,21] line the cleft walls in an arrangement similar to that observed in other helicase structures [22]. Domain 1 contains the Walker A and B motifs that are widely conserved in nucleotide triphosphate hydrolases. The Form 1 ATPγS-soaked crystal exhibited electron density corresponding to the hydrolyzed product ADP, rather than the soaked ATPγS, in the nucleotide-binding pocket (Figure 1b). On the other hand, clear electron density for the bound triphosphate was observed in the Form 2 AMPPCP-soaked crystal (Figure 1c). Regardless of the crystal form and the nucleotide binding, the structures of the Walker-A motif and the surrounding region are very similar to each other.

The ATP-analog AMPPCP is bound to the binding pocket of domain 1, and it participates in several key interactions with the protein: The adenine moiety is surrounded mainly by four hydrophobic residues, Ile21, Phe24, Tyr25, and Leu54, and the two nitrogen atoms hydrogen bond with Gln62, in a bidentate manner. The triphosphate is wrapped up by the Walker A motif (Thr48 to Thr53), which contains the invariant lysine residue (Lys52). The γ-phosphate faces the two acidic residues in the Walker B motif (Asp145 and Glu146). Interestingly, in the Hjm structures, the conformations around the nucleotide binding sites are almost the same, including the side-chain conformations, independently of the nucleotide-binding states. Figure 1d shows a close-up view around the nucleotide binding sites of the three family members. A comparison of the nucleotide binding pocket of Hjm with those in the two Hel308 helicases revealed that the pocket of *A. fulgidus *Hel308 is partly disrupted: In the superimposed structure, the three amino acids of the *A. fulgidus *Hel308 sterically clash with the ATP-analog molecule bound to Hjm (Ile26 with the adenine moiety, and Ala50 and Ala51 with the β-phosphate), indicating that the *A. fulgidus *Hel308 segments should undergo a structural change upon nucleotide binding. On the other hand, the *S. solfataricus *enzyme exhibits a highly similar structure around the nucleotide binding site, and therefore seems to be ready to bind the nucleotide.

The C-terminal region is divided into three domains (domains 3–5). Domain 3 (residues 400–492) has a structural segment similar to the winged-helix (WH) motif. This motif is often used for the recognition and binding of double-stranded DNA (ds DNA) [23]. In the case of Hjm, however, it is unclear whether this segment is important for DNA binding, because the electrostatic potential surface has few notably positive areas in this region. Consistently, in the structure of the *A. fulgidus *Hel308-DNA complex, the corresponding segment was not involved in DNA binding. Domain 4 (residues 492–642) folds into a seven α-helix bundle structure. This fold seems to be unique within this helicase family, as thus far.

The C-terminal domain 5 (residues 643–720) is the smallest and contains the HhH motif. The HhH motif is present in many DNA metabolizing proteins that recognize ssDNA [24]. Indeed, the corresponding element in the *A. fulgidus *Hel308 helicase interacts with DNA [18]. In the case of the *S. solfataricus *and *M. thermautotrophicus *Hel308, this domain exhibited a regulatory function to tune the processivity of its helicase activity as a molecular brake [19,25]. *Pfu*Hjm possesses a PCNA-interacting protein (PIP) box at the C-terminus, which is required for the physical interaction with PCNA, and the unwinding activity of *Pfu*Hjm for the fork-structured DNA is enhanced by PCNA *in vitro *[16]. However, the C-terminal segment was invisible in both the Form 1 and Form 2 crystals, suggesting that this segment is highly mobile.

### The Interaction of *PfuHjm *with DNA is similar to that of the archaeal Hel308 helicase

Based on the *A. fulgidus *Hel308-DNA crystal structure, a DNA unwinding mechanism has been proposed for this helicase [18]. In this mechanism, the central helix of domain 4 acts as the "ratchet" formed by two key amino acid residues (Arg592 and Trp599 of *A. fulgidus *Hel308). These residues form stacking interactions on base moieties of the DNA, thus pushing out 3' tails of unwound DNAs from the tunnel, formed by the domains 1, 3, and 4, toward an exit near domain 5. An interesting feature is that the ratchet helix is located near the conserved helicase motifs, Ia and Ib of domain 1, and IV of domain 2, which are associated with ATPase activity.

*Pfu*Hjm shares 30% and 37% amino acid identity with the *A. fulgidus *and *S. solfataricus *Hel308 helicases, respectively (see Additional file 1: Multiple sequence alignment), and the overall folding of *Pfu*Hjm is very similar to those of those proteins throughout the molecule. *Pfu*Hjm was fitted to the protein of the *A. fulgidus *Hel308-DNA complex, with an rmsd of 2.06 Å, for the corresponding 561 C_a _atoms, while rmsd values for individual fitting of each domain is 1.04, 0.99, 0.90, 1.13, and 1.22 Å, for domains 1 to 5, respectively. This indicates that the spatial arrangements of the five domains significantly differ between *Pfu*Hjm and the DNA-bound *A. fulgidus *Hel308, and hence each domain of *Pfu*Hjm was separately fitted to the corresponding domain of *A. fulgidus *Hel308.

**Figure 2:**
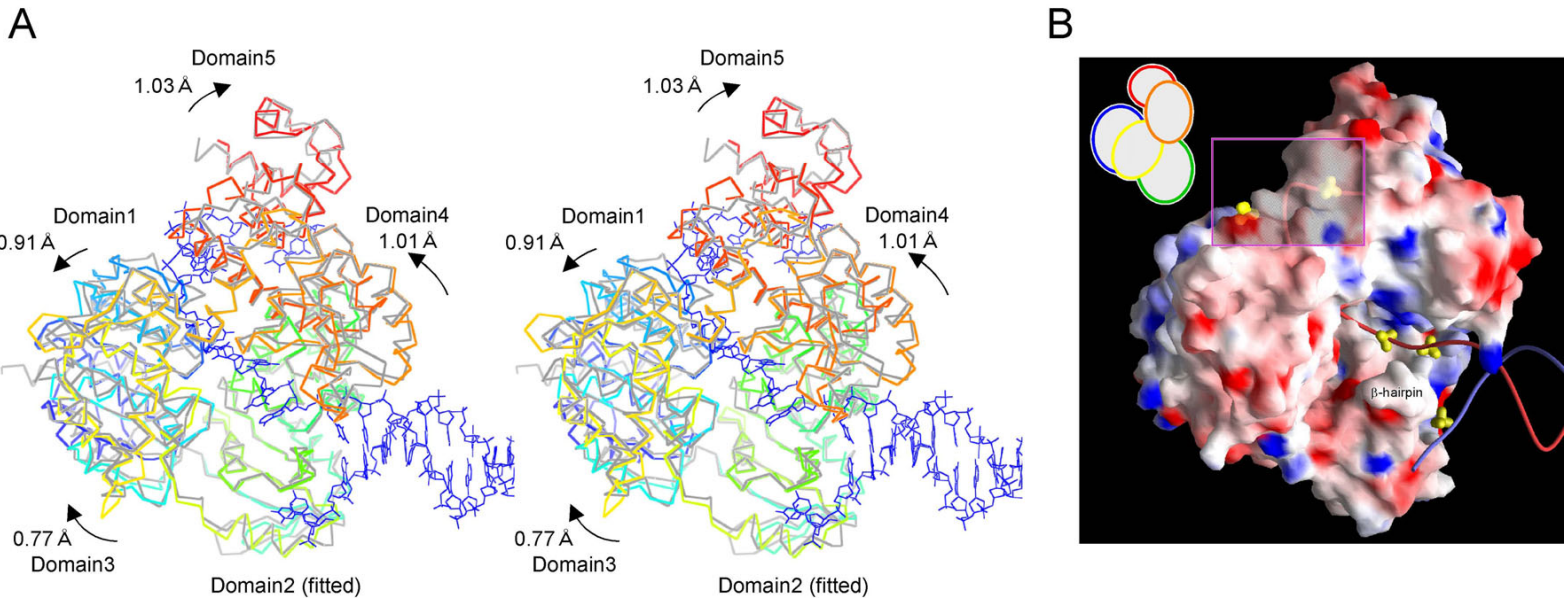
**Structural comparison of *Pfu*Hjm with the *A. fulgidus *Hel308-DNA complex**. (A) Hjm (colored as in Figure 1) was fitted to Hel308 (protein and DNA are colored grey and blue, respectively), by superimposing each domain individually. First, Domain 2 of Hjm was fitted, and then the other domains were separately moved to the best-fit positions. Shift values on the second fitting are indicated. (B) Sulfate ion binding sites in the Hjm Form 1 crystals. The protein is represented as a surface colored by electrostatic potential, calculated by the program GRASP [46]. The DNA structure is that in the Hel308-DNA complex. The boxed region is shown by a transparent surface to show the sulfate ions located inside of the protein.

First, we superimposed *Pfu*Hjm on *A. fulgidus *Hel308 only using domain 2, which recognizes the branch points of the substrate DNA, and then the other four domains were further moved separately to the best fitted positions. The shifts of the second fitting could correspond to the movements of each domain upon DNA binding. The second shifts, defined as the center of mass, were 0.91, 0.77, 1.01, and 1.03 Å for domains 1, 3, 4, and 5, respectively (Figure 2a). Therefore, as suggested previously for *A. fulgidus *Hel308 [18], the domain rearrangement of *Pfu*Hjm should be small upon branched DNA processing. According to the scheme of the helicase-DNA recognition revealed from the *A. fulgidus *Hel308-DNA complex crystal structure, we visually inspected which amino acids interact with DNA in the fitted *Pfu*Hjm-DNA binding model, and found out that such amino acids and their locations are substantially conserved (see Additional file 1: Multiple sequence alignment).

Furthermore, the prominent β-hairpin loop in domain 2, which melts the duplex DNA in *A. fulgidus *Hel308, is shorter by one residue in *Pfu*Hjm. However, the residues that contact the DNA in the *A. fulgidus *Hel308-DNA complex are substantially conserved in the sequence, and thus the protein-DNA interactions at this β-hairpin loop could be quite similar between the two enzymes. In the *Pfu*Hjm crystal structures, several segments exhibit high temperature factors, and the side chain atoms could not be assigned in the electron density maps. Among these, segments 332–335 and 347–351 are located on the possible DNA interacting surfaces. We presume that their conformational flexibility would be important for the continuous DNA translocating and unwinding reaction, which is coupled with ATP binding/hydrolysis.

The Form1 crystals were obtained using ammonium sulfate as a precipitant, and it was found that five sulfate ions were bound to the protein. Notably, all of the sulfate ions lie on possible DNA binding surfaces (Figure 2b). Similarly, it was reported that phosphate ions are bound in the *A. fulgidus *and *S. solfataricus *Hel308 structures [18,19]. Collectively, these results indicate that the sulfate/phosphate ions mimic DNA backbone phosphates. For instance, a sulfate ion is strongly bound to Arg306 and Arg309 in domain 2 of *Pfu*Hjm. Two point mutations (R306A or R309A) in *Pfu*Hjm significantly decreased the DNA binding ability (Fujikane and Ishino, unpublished data).

Taken together, the *Pfu*Hjm structures strongly suggest that this helicase recognize branched DNAs in a similar manner to that in the *A. fulgidus *Hel308-DNA complex. Therefore, it is also likely that the DNA unwinding mechanism is conserved between them.

### Electron microscopy of *PfuHjm *complexed with DNA

**Figure 3:**
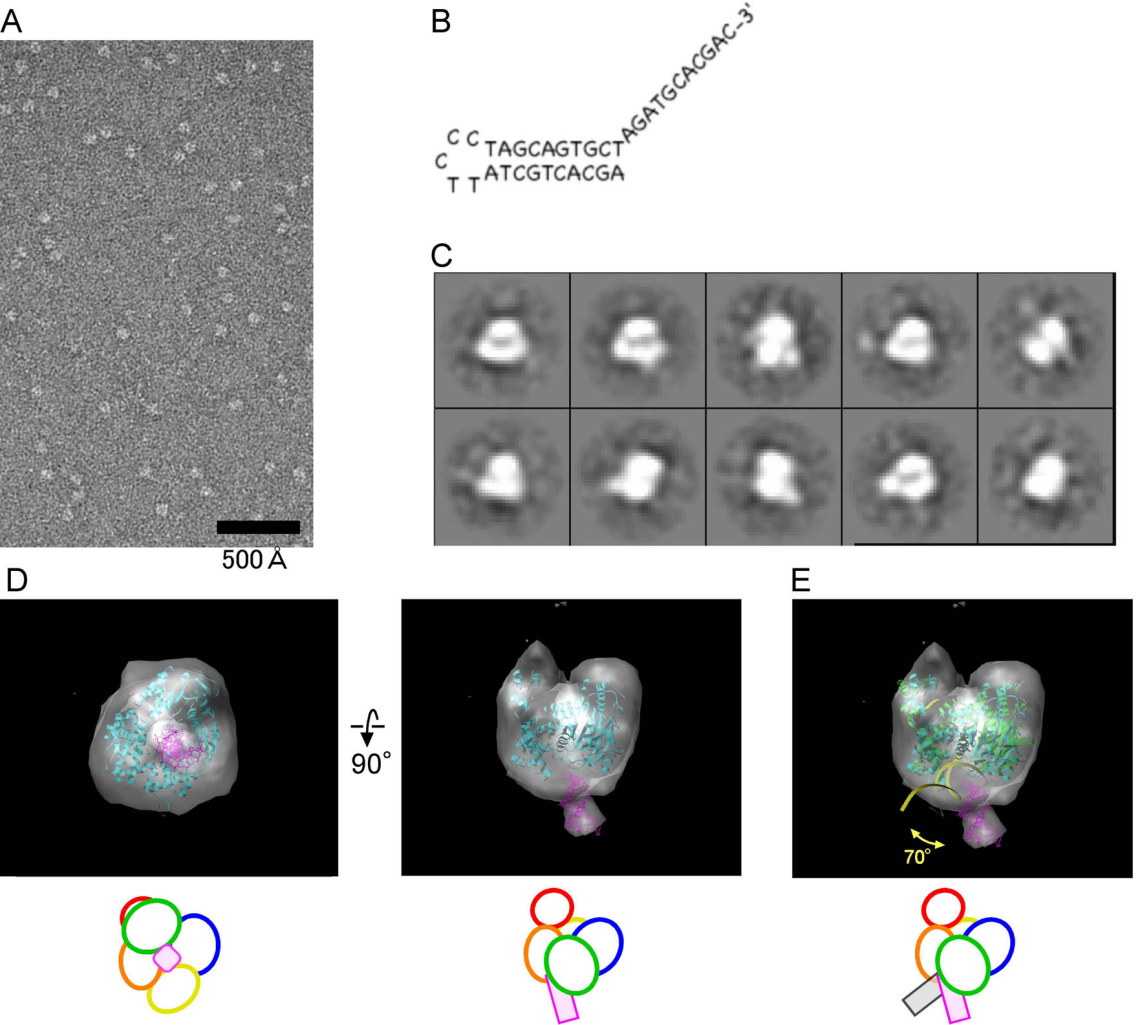
**Electron microscopy of the *Pfu*Hjm-DNA complex**. (A) Representative electron microscopic images of the complex. (B) The sequence and structure of the 3' overhang DNA. (C) Class averages of the Hjm-DNA complex, obtained from 3599 particles. The box size is 204 Å. (D) Single particle 3D reconstruction of the complex. Surface representations of the complex are shown from two orthogonal orientations. The atomic structure of the Hjm protein, shown as a cyan ribbon, is fitted into the density. The double-stranded DNA molecule, colored magenta, is fitted to the protruded region. (E) Comparison with the crystal structure of the *A. fulgidus *Hel308-DNA complex. The *A. fulgidus *binary complex, shown as a green and yellow ribbon, was fitted to the map using the Hel308 protein, and the location of the protruded double-stranded DNA is shown for comparison.

We were not successful in obtaining *Pfu*Hjm DNA complex crystals. Therefore, we used single particle electron microscopy to analyze the structure of a *Pfu*Hjm in complex with a 3' overhang DNA, and indeed, a 3D image was obtained at 23Å resolution (Figure 3). The complex has a main body with a protruded portion. The main body corresponds to *Pfu*Hjm, as the atomic structure of *Pfu*Hjm fits well into the electron density isosurface. Consequently, the protruded portion should correspond to the ds DNA lying outside of the protein molecule. It should be noted that the orientation of the ds DNA is different between the *Pfu*Hjm-DNA EM structure and the *A. fulgidus *Hel308-DNA crystal structure. The ds DNA in our complex is tilted by about 70 degrees, as compared to that in the *A. fulgidus *enzyme complex. The sequence and the secondary structure of DNA used in our study is slightly different from that of the Hel308 complex. However, it is unlikely that this caused the difference in DNA orientations. In fact, the double-stranded region of the DNA substrate, in both of the protein-DNA complexes, weakly interacts with the helicases through minor contacts. For instance, our previous electrophoresis mobility shift assay (EMSA) indicated that the apparent dissociation constant of *Pfu*Hjm against ds DNA was about 5 times higher than those against single-stranded or Y-shaped DNA [16]. Thus, the ds DNA may have happened to be fixed at the distinct positions, because crystallographic and EM analyses target different states of protein or protein-DNA complexes.

### Comparison with other helicases

Apart from the Hel308 helicases, Hjm is closest to a bacterial RecQ helicase (1oywA) [26] in its N-terminal region (domains 1 and 2). On the other hand, the C-terminal halves of Hjm and the archaeal Hel308s adopt unique folds. However, we could detect local fold similarity of domain 3 to transcriptional factors (Arg repressor, 1aoy [27], and transcription initiation factor IIF, 1onvA [28]). Likewise domain 4 shares local similarity to the signal recognition particle protein (1hq1A) [29], while the C-terminal domain 5 shares similarity to DNA excision repair protein (2a1jB) [30] and HJ DNA binding protein (1d8lA) [31].

**Figure 4:**
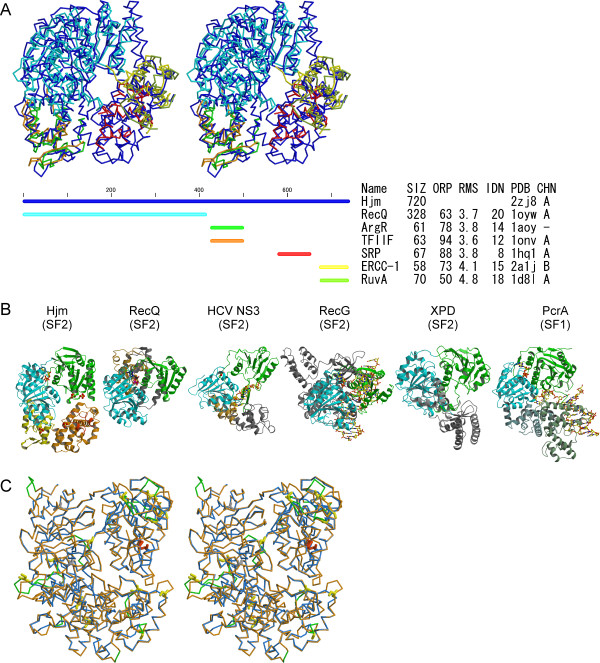
**Schematic view of the structural alignment**. (A) Domain structure conservation of *Pfu*Hjm with relevant DNA binding proteins. SIZ, ORP, RMS, and IDN are the number of residues aligned, percent superposed residues (% overlap), RMSD (Å), and sequence identity (%) of each domain to Hjm, respectively. PDB and CHN are the PDB code and chain ID of the solutions, respectively. Abbreviations, Hjm; *Pfu*Hjm, RecQ; *E. coli *RecQ, ArgR; *E. coli *Arginine repressor, TFIIF; Transcription initiation factor IIF, SRP; single recognition particle protein, ERCC-1; Human DNA endonuclease ERCC-1, RuvA; *E. coli *Holliday junction binding protein RuvA. (B) Structure comparison of Hjm with other relevant proteins. Structures are aligned using the two well-conserved helicase domains (cyan and green), to highlight the variation of the other domains and those orientation. (C) Superposition of *Pfu*Hjm (blue) and the homology model of the human DNA polymerase Θ (orange) helicase domain, shown in stereo figure. The major-insertions (with more than two residues) of the DNA polymerase Θ were colored green. The insertions were localized to the peripherals of the molecule, and the central crafts of the proteins are mostly intact. The seventeen cysteine residues of the DNA polymerase Θ are shown in stick models, and colored according to their possible characteristics (buried, grey; exposed, yellow; disulfide bond, red)

The Hjm structure appears to be composed of a unique combination of the domains used for DNA/RNA-binding or processing. The overall structural comparison among the SF2 helicases is shown in Figure 4b. When these structures are aligned using the well-conserved helicase domains, the configurations of the other domains are quite variable. This indicates that these enzymes share the two helicase domains that are fundamental for the helicase activity, while the structural and spatial arrangements of the other domains are designed to correspond to their individual DNA unwinding mechanisms and substrate specificities.

### Homology modeling of the human PolΘ helicase domain indicates structural and functional similarity to *PfuHjm*

The DNA metabolizing proteins from archaea are both structurally and functionally similar to those from eukaryote, and therefore, the structures of archaeal proteins are useful to understand the complicated DNA transaction mechanisms in eukaryotes. In this study, we showed that the 3D structure of *Pfu*Hjm is similar to those of the *A. fulgidus *and *S. solfataricus *Hel308 helicases, implying that these structural features could be extended to this helicase family, which includes the human PolΘ and Hel308 and Drosophila Mus308 proteins. Human PolΘ is A-family DNA polymerase and works in translesion DNA synthesis [32,33]. This protein is unique because it has both helicase and DNA polymerase domains on a single polypeptide chain. A homology model of the helicase domain of human PolΘ, which was built using the program MOE (Ryoka Systems Inc.), is highly similar to the *Pfu*Hjm and Hel308 helicases (Fig. 4c; also see Additional file 2: Homology model of the human DNA polymeraseΘ helicase domain). The model seems to be reasonable in that, as in the case of *Pfu*Hjm, the putative DNA-interacting segments are both sequentially and spatially conserved in the human PolΘ helicase domain. In this domain, PolΘ contains seventeen cysteine residues that are not present in *Pfu*Hjm. The homology model indicates that twelve cysteine residues are exposed to the solvent, and that two of them form a disulfide linkage in a region corresponding to domain 2 of *Pfu*Hjm. Furthermore, several cysteine residues are conserved in PolΘ helicase domains in eukaryotes other than human (see Additional file 1: Multiple sequence alignment). It is tempting to speculate that these cysteines are used for sensing oxidative stress, because a genetic analysis showed that vertebrate PolΘ gene-deficient cells exhibited hypersensitivity to oxidative base damage induced by H_2_O_2 _[34].

## Conclusion

We determined the high-resolution crystal structures of the archaeal SF-2 helicase, *Pfu*Hjm. Although we could not obtain the protein-DNA complex structures, in comparison with the previously reported Hel308-DNA complex, the 3D EM image of the Hjm-DNA complex suggested that the two helicases unwind DNA by essentially the same mechanism. Furthermore, homology modeling of the human DNA polymerase Θ helicase domain strongly suggested the structural conservation across the domains of life.

As suggested by the structural study of the *A. fulgidus *Hel308-DNA complex, the DNA unwinding mechanism itself may differ between the Hel308 family proteins and *E. coli *RecQ and related proteins, because of the lack of the β-hairpin loop. However, accumulating biochemical evidence suggests that *Pfu*Hjm, and probably the closely-related archaeal proteins, are the functional counterparts of the *E. coli *RecQ helicase.

## Methods

### Protein expression and purification

The recombinant *Pfu*Hjm protein was produced and purified as described previously [15]. The gene encoding the protein was cloned into the pET21d vector, and the constructed plasmid, pHJM100, was introduced into *E. coli *BL21 codonPlusTM (DE3)-RIL cells (Stratagene). The transformed cells were grown in LB medium containing 50 μg/mL ampicillin and 34 μg/mL chloramphenicol at 37°C to an OD_600 _of 0.35, and then protein expression was induced by 1 mM IPTG for 5 h. The cells were harvested and disrupted by sonication in buffer A (50 mM Tris-HCl, pH 8.0, 0.5 M NaCl, 0.5 mM EDTA, 1 mM DTT, and 10% glycerol). The soluble fraction was collected by centrifugation (12 000 *g*, 15 min) and then was incubated at 80°C for 20 min. Polyethylenimine was added to the supernatant to a final concentration of 0.15% (v/v), to remove the nucleic acids. The soluble fraction was clarified by centrifugation and precipitated by 80%-saturated ammonium sulfate. The proteins were resuspended in buffer B (50 mM Tris-HCl, pH 8.0, 1.25 M (NH_4_)_2_SO_4_, 0.5 mM EDTA, 1 mM DTT, and 10% glycerol), loaded onto a hydrophobic column (HiTrap Butyl, GE Healthcare), and eluted with H_2_O. The pooled fraction was dialyzed against buffer C (10 mM K-phosphate, 7 mM β-mercaptoethanol, 0.01 mM CaCl_2_, and 10% glycerol) and was loaded onto a CHT-II hydroxyapatite column (Bio-Rad), which was developed with a linear gradient of 0.01 to 1 M K-phosphate. The fraction pool containing the *Pfu*Hjm protein of interest was subsequently dialyzed against buffer D (50 mM Tris-HCl, pH 8.0, 0.5 mM EDTA, 1 mM DTT, and 10% glycerol), and was loaded onto an anion exchange column (MonoQ 5/5, GE Healthcare). The column was developed with a 0 to 1 M NaCl linear gradient, and the purified protein was eluted at 0.32–0.37 M NaCl. The purified protein was concentrated to 8 mg/ml for crystallization. The calculated extinction coefficient of 101,190 M^-1^cm^-1 ^at 280 nm was used for the determination of the protein concentration. To prepare the selenomethionine (SeMet) derivative of *Pfu*Hjm, pHJM100 was transformed into the methionine auxotrophic strain *E. coli *BL21(DE3) Codonplus RIL-X (Stratagene). The SeMet derivative was expressed by IPTG induction in a minimal medium containing seleno-L-methionine at a final concentration of 25 μg/ml, and was purified using the same procedure as for the wild type protein.

### Crystallization, data collection, and model refinement

*Pfu*Hjm was crystallized by the hanging drop vapor diffusion technique with the micro-seeding at 293 K. The first diffraction quality crystals (Form 1) were obtained using a reservoir containing 100 mM citrate (pH 5.0) and 1.6 M ammonium sulfate. The crystals belonged to the space group *C*2, with unit cell constants *a *= 118.6 Å, *b *= 85.0 Å, *c *= 95.0 Å, and β = 121.0°, and contained one Hjm molecule per asymmetric unit. The SeMet protein was crystallized under the same conditions as for the wild-type Hjm. Tantalum (Ta_6_Br_14_)- and platinum (K_2_PtCl_4_)- derivatized crystals were prepared by soaking. ATPγS-soaked crystals were prepared by soaking native crystals in reservoir solution containing 1 mM ATPγS. Crystals were harvested with the reservoir solution containing 20% (v/v) glycerol for X-ray diffraction data collection at 100 K. Data sets of the native crystal and a Pt-derivative were collected on BL-6B of the Photon Factory, Tsukuba, Japan. The Ta derivative data were collected on BL40-B2, and those for the ATPγS-soaked crystal and the Se-Met derivative were obtained on BL41-XU of SPring-8 (Harima, Japan). Data sets were processed by DENZO/SCALEPACK or the HKL2000 package [35].

The structure was determined by the MIRAS method. All the heavy atom sites were located on isomorphous Patterson maps, and the heavy atom parameters were refined by the program SHARP [36]. The experimental phases were improved by density modification techniques, with the programs DM and SOLOMON in the CCP4 suite [37]. The initial atomic model was built, based on this modified map, with the program O [38]. About 70% of the amino acid residues were located using the modified map. The combination of the experimental MIRAS phases with those calculated from a partial model further improved the quality of the electron density map, leading to the construction of the other parts. Crystallographic refinement was performed with the program CNS [39]. The final model of the Form 1 apo crystal consisted of 660 amino acid residues, except for the disordered region (mainly the C-terminal 60 residues). The structure of the ATPγS-soaked crystal was determined by using the apo-form as the initial model, and was refined to convergence. Careful inspection of the electron density maps revealed that the bound nucleotide was the hydrolyzed product ADP, rather than the soaked ATP-analog.

The second crystals (Form 2) were obtained under different crystallization conditions, using a reservoir solution containing 80 mM Tris-HCl (pH 8.5), 160 mM CaCl_2_, and 11% (w/v) PEG4000. The micro-seeding technique was also used to obtain diffraction quality crystals. These crystals also belonged the space group *C*2, as did Form 1, but had significantly different unit cell constants (*a *= 122.3 Å, *b *= 81.2 Å, *c *= 85.2 Å, and β = 111.9°), suggesting distinct crystal packing. The complex with AMPPCP was prepared by soaking the Form 2 apo crystals into reservoir solution containing 0.5 mM AMPPCP. Diffraction data sets for the Form 2 apo crystal were collected at 100 K on BL38-B1 of SPring-8, and those for the AMPPCP complex crystal were collected at BL-6B of the Photon Factory. These structures were determined by molecular replacement, using the program CNS and the Form 1 apo structure as a probe. The Form 2 structures are better ordered in the crystals, and the almost the entire molecule, except for the C-terminal 20 residues with the PIP-box sequence, was visible in the electron density map. Crystallographic refinements were reiterated to obtain satisfactory convergence. All of the crystallographic statistics are summarized in Table 1. The atomic coordinates have been deposited in the Protein Data Bank, under the accession codes 2ZJ2, 2ZJ5, 2ZJ8, and 2ZJA, for the Form 1 apo, Form 1 ADP complex, Form 2 apo, and Form 2 AMPPCP complex, respectively.

### Electron microscopy

The 3' overhang DNA was prepared by forming a hairpin structure from a synthetic oligonucleotide (5'- AGCACTGCTATTCCCTAGCAGTGCTAGATGCACGAC-3'). The Hjm protein was mixed with DNA (1:1 protein/DNA ratio) and was incubated in a buffer containing 50 mM Tris-HCl pH8.0, 0.15 M NaCl, 0.5 mM EDTA, 1 mM DTT, and 10% glycerol, at room temperature for 20 min. The complex was purified by gel filtration chromatography on a Superdex 200 PC 3.2/30 column (GE Healthcare), using a SMART system (GE Healthcare). An aliquot of the complex solution was applied to a carbon support film, and was negatively stained with 2% uranyl acetate. The specimens were examined with a JEM 1010 electron microscope (JEOL), operated at an accelerating voltage of 100 kV. Images were recorded by BioScan CCD camera (Gatan). A minimum dose system (MDS) was used to reduce the electron radiation damage of the sample. The step size of a pixel of the image was calibrated to be 5.1 Å, using TMV as a reference sample. Image processing was performed using the software packages EMAN [40] and IMAGIC [41]. Individual particle images were boxed out, using the GUI-based program boxer in EMAN. The class average images of the Hjm-DNA complexes were obtained by several cycles of a multireference alignment and classification procedure for image sets. The programs in IMAGIC were used to calculate these class averages. The initial 3D map was obtained by common-line method and subsequent iterative refinement was performed using REFINE routine of EMAN. The resolution of the 3D map was estimated by the 0.5 criterion of the Fourier shell correlation. The visualization of the 3D map and fitting of the crystal structure into the map were performed, using the Chimera software [42].

### Homology modeling

The homology model of the helicase-like domain of human DNA polymerase Θ (UniProt code Q6VMB5) was constructed by using the Homology module of the MOE application (Ryouka Systems Inc.), which was based on the methods of Levitt [43] and Fechteler et al. [44].

## Authors' contributions

TO carried out the crystallization and structure determination, and wrote the manuscript. HO carried out the protein expression, purification, and crystallization. K Mayanagi carried out electron microscopy and helped to write the manuscript. TS designed and performed the homology modeling and helped to write the manuscript. K Matoba assisted with electron microscopy. RF carried out the biochemical experiments and participated in the discussions of the study. YI and K Morikawa conceived of the study and developed the manuscript. All authors read and approved the final manuscript.

## Supplementary Material

Additional file 1 **Multiple sequence alignment**. Hjm helicases and the homologues of *Pyrococcus furiosus *(Euryarchaeota, Hel_Pfu, O73946|HELS_PYRFU), *Archaeoglobus fulgidus *(Euryarchaeota, Hel_Afu, NC_000917.1), *Methanococcus vannielii *(Euryarchaeota, Hel_Mva, A2UUA3|A2UUA3_METVA), *Sulfolobus solfataricus *(Crenarchaeote, Hel_Sso, Q97VY9|HELS_SULSO), and *Methanopyrus kandleri *(Euryarchaeota, Hel_Mka, Q8TGZ1|Q8TGZ1_METKA), and DNA polymerase Θ s and the homologues of *Homo sapiens *(human, PolQ_Has, Q96SE4|Q96SE4_HUMAN), *Mus musculus *(mouse, PolQ_Mmu, Q80XB7|Q80XB7_MOUSE), *Aspergillus oryzae *(fungus, PolQ_Aor, Q2UKG1|Q2UKG1_ASPOR), *Aedes aegypti *(mosquito, PolQ_Aae, Q178I3|Q178I3_AEDAE), and *Trypanosoma brucei *(protista, PolQ_Tbr, Q57YX8|Q57YX8_9TRYP) are aligned (in parentheses are the organism common name, name in the alignment, and UniProt or Refseq code). The presented sequences were selected from a total of 341 sequences as the representatives of clusters. The conservative Cys residues among the DNA polymerases are indicated by + (conserved among the shown sequences: Cys322 and Cys477) or # (conserved except for protista: Cys232, Cys483, Cys629, Cys651, and Cys289) below the alignment. Amino acid residues are colored according to their characteristics (aromatic, cyan; hydrophobic, green; basic, brown; ambivalent, orange). Secondary structural elements are indicated on the top of the sequences. DNA binding residues in the *A. fulgidus *hel308-DNA complex are depicted by triangles. Putative disulfide bond-forming residues in the human PolΘ helicase domain model are colored yellow-. The figure was prepared with ClustalX. Click here for file

Additional file 2 **Homology model of the human DNA polymerase Θ helicase domain**. The model was built using the program MOE (Ryoka Systems Inc.) Click here for file
