# Supplementary material for: Atomic structures and functional implications of the archaeal RecQ-like helicase Hjm
Source: BMC Struct Biol. 2009 Jan 22;9:2. doi: 10.1186/1472-6807-9-2 (PMC2636818; doi:10.1186/1472-6807-9-2)
Supplement: Additional file 1 — Multiple sequence alignment. Hjm helicases and the homologues of Pyrococcus furiosus (Euryarchaeota, Hel_Pfu, O73946|HELS_PYRFU), Archaeoglobus fulgidus (Euryarchaeota, Hel_Afu, NC_000917.1), Methanococcus vannielii (Euryarchaeota, Hel_Mva, A2UUA3|A2UUA3_METVA), Sulfolobus solfataricus (Crenarchaeote, Hel_Sso, Q97VY9|HELS_SULSO), and Methanopyrus kandleri (Euryarchaeota, Hel_Mka, Q8TGZ1|Q8TGZ1_METKA), and DNA polymerase Θ s and the homologues of Homo sapiens (human, PolQ_Has, Q96SE4|Q96SE4_HUMAN), Mus musculus (mouse, PolQ_Mmu, Q80XB7|Q80XB7_MOUSE), Aspergillus oryzae (fungus, PolQ_Aor, Q2UKG1|Q2UKG1_ASPOR), Aedes aegypti (mosquito, PolQ_Aae, Q178I3|Q178I3_AEDAE), and Trypanosoma brucei (protista, PolQ_Tbr, Q57YX8|Q57YX8_9TRYP) are aligned (in parentheses are the organism common name, name in the alignment, and UniProt or Refseq code). The presented sequences were selected from a total of 341 sequences as the representatives of clusters. The conservative Cys residues among the DNA polymerases are indicated by + (conserved among the shown sequences: Cys322 and Cys477) or # (conserved except for protista: Cys232, Cys483, Cys629, Cys651, and Cys289) below the alignment. Amino acid residues are colored according to their characteristics (aromatic, cyan; hydrophobic, green; basic, brown; ambivalent, orange). Secondary structural elements are indicated on the top of the sequences. DNA binding residues in the A. fulgidus hel308-DNA complex are depicted by triangles. Putative disulfide bond-forming residues in the human PolΘ helicase domain model are colored yellow-. The figure was prepared with ClustalX. [file 1472-6807-9-2-S1.pdf]

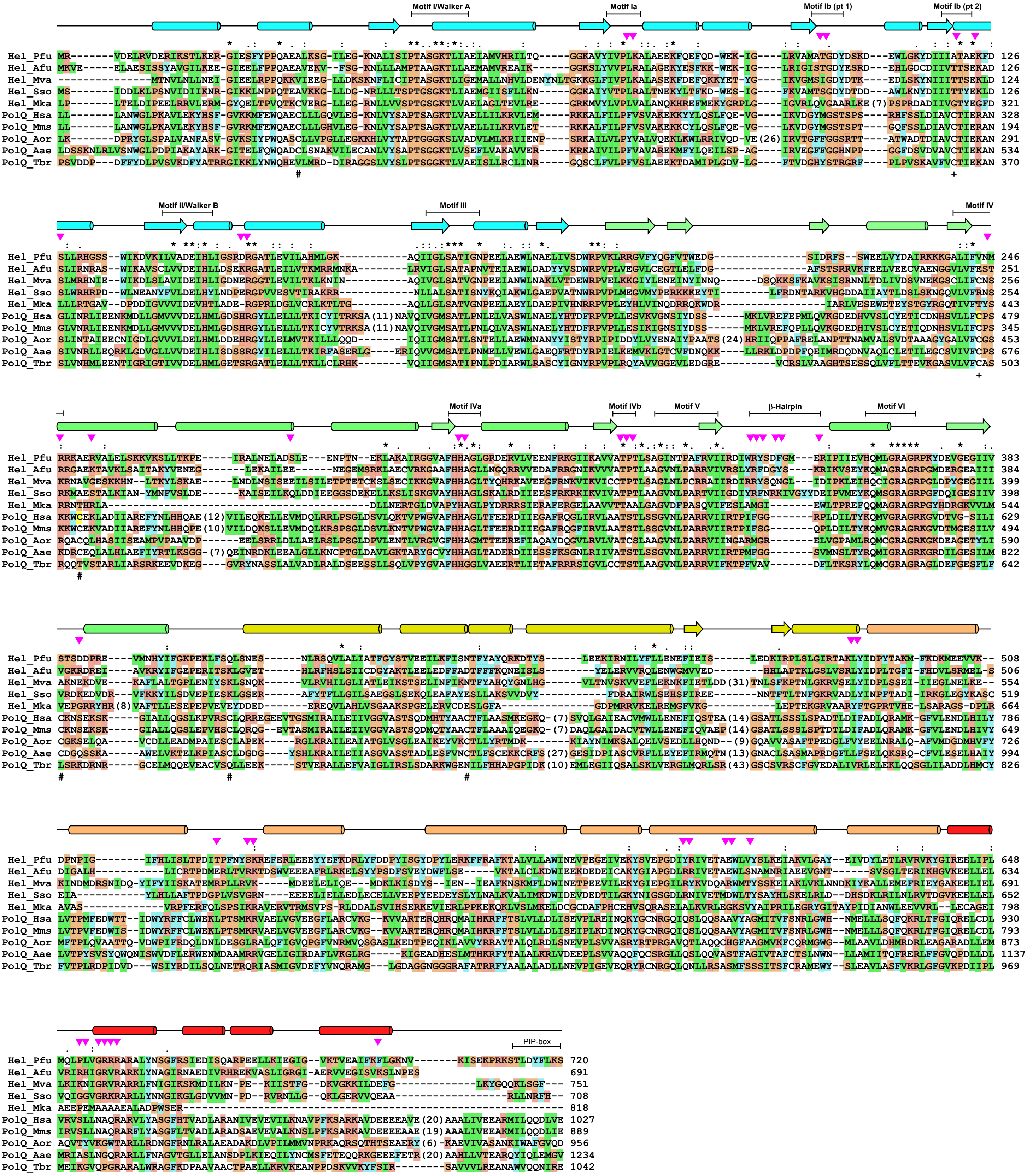
Motif I/Walker A
Motif Ia
Motif Ib (pt 1)
Motif Ib (pt 2)
Hel_Pfu MR-------VDELRVDERIKSTLKER-GIESFYPPQAEALKSG-ILEG-KNALISIPTASGKTLIAEIAMVHRILTQ-----GGKAVYIVPLKALAEEKFQEFQD-WEK-IG----LRVAMATGDYDSKD---EWLGKYDIIIATAEKFD 126
Hel_Afu MKVE------ELAESISSYAVGILKEE-GIEELFPPQAEAVEKV--FSG-KNLLLAMPTAAGKTLLAEMAMVREAIK-----GGKSLYVVPLRALAGEKYESFKK-WEK-IG----LRIGISTGDYESRD---EHLGDCDIIVTTSEKAD 126
Hel_Mva ---------------MTNVLNLLNEI-GIEELRPPQKKVIEEG-LLDKSKNFLICIPTASGKTLIGEMALLNHVLDENYNLTGKKGLFIVPLKALASEKFDEFQKKYET-YG----IKVGMSIGDYDTK----EDLSKYNIIITTSEKLD 124
Hel_Sso MS-------IDDLKLPSNVIDIIKNR-GIKKLNPPQTEAVKKG-LLDG-NRLLLTSPTGSGKTLIAEMGIISFLLKN-----GGKAIYVTPLRALTNEKYLTFKD-WES-IG----FKVAMTSGDYDTDD---AWLKNYDIIITTYEKLD 126
Hel_Mka LP-------LTELDIPEELRRVLERM-GYQELTPVQTKCVERG-LLEG-RNLLVVSPTGSGKTLVAELAGLTEVLRE-----GRKMVYLVPLVALANQKHREFMEKYGRPLG----IGVRLQVGAARLKE(7)PSPRDADIIVGTYEGFD 321
PolQ_Hsa LL-------LANWGLPKAVLEKYHSF-GVKKMFEWQAECLLLGQVLEG-KNLVYSAPTSAGKTLVAELLILKRVLEM-----RKKALFILPFVSVAKEKKYYLQSLFQE-VG----IKVDGYMGSTSPS----RHFSSLDIAVCTIERAN 328
PolQ_Mms LL-------LANWGLPKAVLEKYHSF-GVRKMFEWQAECLLLGHVLEG-KNLVYSAPTSAGKTLVAELLILKRVLET-----RKKALFILPFVSVAKEKKCYLQSLFQE-VG----LKVDGYMGSTSPT----GQFSSLDIAVCTIERAN 194
PolQ_Aor LK-------DPRYGLSPALVANFASV-GVKSIYPWQASCLLVPGLLEGKKHLVYTAPTGGGKSLVADVLMLKRIIENP----SRKAILVLPYVALVQEKLKWLRRIVQD-VE(26)IRVTGFFGGSRTT----ATWADTDIAVCTIEKAN 291
PolQ_Aae LDSSKNLRLVSNWGLPDPIAKAYARK-GITELFQWQADCLSNAKVILECANLVYSAPTSGGKTLVSEFLVAKAVVER-----KRKAIVILPFVAVAREKMFYLQEILSP-AG----IRVEGFFGGHNPP----GGFDSVDVAVCTIEKAN 534
PolQ_Tbr PSVDDP---DFFYDLPVSVKDFYATRRGIKKLYNWQHEVLMRD-DIRAGGSLVYSLPTSGGKTLVAEISLLRCLINR-----GQSCLFVLPFVSLAEEKTDAMIPLGDV-LG----FTVDGHYSTRGRF-----PLPVSKAVFVCTIEKAN 370
Motif II/Walker B
Motif III
Motif IV
Hel_Pfu SLLRHGSS--WIKDVKILVADEIHLIGSRDRGATLEVILAHMLGK-----------AQIIGLSATIGNPEELAEWLNAELIVSDWRPVKLRRGVFYQGFVTWEDG---------------SIDRFS--SWEELVYDAIRKKKGALIFVNM 246
Hel_Afu SLIRNRAS--WIKAVSCLVVDEIHLLDSEKRGATLEILVTKMRRMNKA---------LRVIGLSATAPNVTEIAEWLDADYYVSDWRPVPLVEGVLCEGTLELFDG-------------AFSTSRRVKFEELVEECVAENGGVLVFEST 251
Hel_Mva SLMRHNIE--WIKDLSLAVIDEIHLIGDNERGGTLEVILTKLKNIN----------AQIVGLSATVGNPEEIANWLNAKLVTDEWRPVELKKGIYLENEINYINNQ------DSQKKSFKAVKSISRNNLTDLIVDSVNEKGSCLIFCNS 256
Hel_Sso SLWRHRPD--WLNEANYFVLDELHYLNDPERGPVVESVTIRAKRR-----------NLLALSATISNYKQIAKWLGAEPVATNWRPVPLMEGVMYPERKKKEYTI--------LFRDNTARKVHGDDAIIAYTLDSLSKNGQVLVFRNS 254
Hel_Mka LLLRTGAV--DPDDIGVVVIDEVHTLADE-RGPRLDGLVCRLRKLTG---------AQLLGLSATVGNPEELAEYLDAEPIVHNRRPVPLEYHLVINQDRRQKWDR---------------IARLVESEWETEYSTGYRGQTIVFTYS 443
PolQ_Hsa GLINRLIEENKMDLLGMVVVDELHMLGDSHRGYLLELLLTKICYITRKSA(11)NAVQIVGMSATLPNLELVASWLNAELYHTDFRPVPLLESVKVGNSIYDSS------MKLVREFEPMLQVKGDEDHVVSLCYETICDNHSVLLFCPS 479
PolQ_Mms GLVNRLIEENKMDLLGMVVVDELHMLGDSHRGYLLELLLTKICYVTRKSA(11)NAVQIVGMSATLPNLQLVASWLNAELYHTDFRPVPLLESIKIGNSIYDSS------MKLVREFQPLLQVKGDEDHIVSLCYETIQDNHSVLLFCPS 345
PolQ_Aor SLINTAIEECNIGDLGVVVLDELHMLDDEHRGYLLELMVTKILLLQQD--------ISIIGMSATLSNTELLAEWMNANYYISTYRPIPIDDYLVYENAIYPAATS(24)HRIIQPPAFRELANPTTNAMVALSVDTAAAGYGALVFCGS 453
PolQ_Aae SIVNRLLEQRKLGDVGLLVVDEIHLISDSSRGYILELLLTKIRFASERLG----ERIQVVGMSATLPNMELLVEWLGAEQFRTDYRPIELKEMVKLGTCVFDNQKK----LLRKLDPDPFQEIMRDQDNVAQLCLETILEGCSVIVFCPS 676
PolQ_Tbr SLVNHMLEENTIGRIGTIVVDELHMLGETSRGATLELLLTKLLCLRHK---------VQIIGMSATIPNLPDIARWLRASCYIGNYRPVPLRQYAVVGGEVLEDGRE---------VCRSLVAAGHTSESSQLVFLTTEVKGASVLVFCAS 503
Motif IVa
Motif IVb
Motif V
β-Hairpin
Motif VI
Hel_Pfu RRKAERVALELSKKVKSLLTKPE-----IRALNELADSLE---ENPTN--EKLAKAIRGGVAFHHAGLGRDERVLVEENFRKGIIKAVVATPTLSAGINTPAFRVIIRDIWRYSDFGM---ERIPIIEVHQMLGRAGRPKYDEVGEGIIV 383
Hel_Afu RRGAEKTAVKLSAITAKYVENEG-------LEKAILEE-----NEGEMSRKLAECVRKGAAFHHAGLLNGQRRVVEDAFRRGNIKVVVATPTLAAGVNLPARRVIVRSLYRFDGYS---KRIKVSEYKQMAGRAGRPGMDERGEAIII 384
Hel_Mva KRNAVGESKKHN--LTKYLSKAE-----LNDLNSISEEILSILETPTETCKSLSECIKKGVAFHHAGLTYQHRKAVEEGFRNKVIKVICCTPTLSAGLNLPCRRAIIRDIRRYSQNGL---IDIPKLEIHQCIGRAGRPGLDPYGEGIIL 399
Hel_Sso RKMAESTALKIAN-YMNFVSLDE------KAISEILKQLDDIEEGGSDEKELLKSLISKGVAYHHAGLSKALRDIIEESFRKRKIKVIVATPTLAAGVNLPARTVIIGDIYRFNRKIVGYYDEIPVMEYKQMSGRAGRPGFDQIGESIIV 398
Hel_Mka RRNTHRLA-------------------------------------------DLLNERTGLDVAPYHAGLPYDRRRSIERAFERGELAAVVTTAALGAGVDFPASQVIFESLAMGI-------EWLTPREFEQMAGRAGRPGYHDRGKVVLM 544
PolQ_Hsa KKWCEKLADIIAREFYNLHHQAE(12)VILEQKELLEVMDQLRRLPSGLDSVLQKTVPWGVAFHHAGLTFEERDIIEGAFRQGLIRVLAATSTLSSGVNLPARRVIIRTPIFGG-------RPLDILTYKQMVGRAGRKGVDTVG-SILI 629
PolQ_Mms KKWCEKVADIIAREFYNLHHQPE(10)VILDQKSLLEVMDQLKRSPSGLDSVLKNTVPWGVAFHHAGLTFEERDIIEGAFRQGFIRVLAATSTLSSGVNLPARRVIIRTPIFSG-------QPLDILTYKQMVGRAGRKGVDTMGESILV 494
PolQ_Aor RQACQLHASIISEAMPVPAAVDP------EELSRRLDLLAELRSLPSGLDPVLENTLVRGVGFHHAGMTTEEREFIAQAYDQGVLRVLVATCSLAAGVNLPARRVIINGARMGR-------ELVGPAMLRQMCGRAGRKGKDEAGETYLI 590
PolQ_Aae KDRCEQLALHLAEFIYRTLKSGG-(7)QEINRDKLEEALGLLKNCPTGLDAVLGKTARYGCVYHHAGLTADERDIIESSFKSGNLRIIVATSTLSSGVNLPARRVIIRTPMFGG-------SVMNSLTYRQMIGRAGRKGRDILGESILM 822
PolQ_Tbr RQQTVSTARLIARSRKEEVDKEG----GVRYNASSLALVADLRALDSEESSLLSQLVPYGVAFHHGGLVAEERTLIETAFRRRSIGVLCCTSTLAAGVNLPARRVIFKTPFVAV-------DFLTKSRYLQMCGRAGRAGLDEFGESFLF 642
Hel_Pfu STSDDPRE----VMNHYIFGKPEKLFSQLSNES------NLRSQVLALIATFGYSTVEEILKFISNTFYAYQRKDTYS------LEEKIRNILYFLLENEFIEIS------LEDKIRPLSLGIRTAKLYIDPYTAKM-FKDKMEEVVK-- 508
Hel_Afu VGKRDREI----AVKRYIFGEPERITSKLGVET------HLRFHSLSIICDGYAKTLEELEDFFADTFFFKQNEISLS--------YELERVVRQLENWGMVVED--------HHLAPTKLGSLVSRLYIDPLTGFI-FHDVLSRMEL-S 506
Hel_Mva AKNEKDVE----KAFLALTGPLENIYSKLSNQK------VLRVHILGLIATLEIKSTSELINFIKNTFYAHQYGNLHG------VLTNVSKVVEFLEKNKFIETLDD(31)TNLSFKPTNLGKRVSELYIDPLSSEI-IIEGLNELKE-- 554
Hel_Sso VRDKEDVDR----VFKKYILSDVEPIESKLGSER------AFYTFLLGILSAEGSLSEKQLEAFAYESLLAKSVVDVY----------FDRAIRWLSEHSFIREE-------NNTFTLTNFGKRVADLYINPFTADI-IRKGLEGYKASC 519
Hel_Mka VEPGRRYHR(8)VAFTLLESEPEPVEVEYDDED-------EREQVLAHLVSGAAKSPGELERVCDESLGFA---------------GDPMRRVKELREMGFVKG---------LEPTEKGRVAARYFTGPRTVHE-LSARAGS-DPLR 664
PolQ_Hsa CKNSEKSK-----GIALLQGSLKPVRSCLQRREGEEVTGSMIRAILEIIVGGVASTSQDMHTYAACTFLAASMKEGKQ-(7)SVQLGAIEACVMWLLENEFIQSTEA(14)GSATLSSLSPADTLDIFADLQRAMK-GFVLENDLHILY 786
PolQ_Mms CKNSEKSK-----GIALLQGSLEPVHSCLQRQG-EVTASMIRAILEIIVGGVASTSQDMQTYAACTFLAAAIQEGKQ-(7)DAQLGAIDACVTWLLENEFIQVAEP(14)GSATLSSLSPTDTLDIFADLQRAMK-GFVLENDLHIVY 649
PolQ_Aor CGKSELQA-----VCDLLEADMPAIESCLAPEK-----RGLKRAILEAIATGLVSGLEAIKEYVKCTLLYRTMDK--------KIAYNIMKSALQELVSEDLLHQND--(9)GQAVVASAFTPEDGLFVYEELNRALQ-AFVMDGDMHVFY 726
PolQ_Aae CDGQSSKA-----AWELVKTELKPIASCLDGDG----YSHLKRAILEIIASGVASSTADLESFVNCTLFSCEKKCRFS(27)GESIDPIASCVRFLLEYEFIRMQTN(13)GNACLSASMAPRDGFLLFSELQKSRQ-CFVLESELHAIY 994
PolQ_Tbr LSRKDRNR-----GCELMQQEVEACVSQLLEEK-----STVERALLEFVAIGLIRSLSDARKWGENILFHHAPGPIDK(10)EMLEGIIQSALSKLVERGLMQRLSR(43)GSCSVRSCFGVEDALIVRLELEKLQQSGLILADDLHMCY 826
Hel_Pfu DPNPIG--------IFHLISLTPDITPFNYSKREFERLEEEYYEFKDRLYFDDPYISGYDPYLERKFFRAFKTALVLLAWINEVPEGEIVEKYSVEPGDIYRIVETAEWLVYSLKEIAKVLGAY--EIVDYLETLRVRVKYGIREELIPL 648
Hel_Afu DIGALH----------LICRTPDMERLTVRKTDSWVEEEAFRLRKELSYYPSDFSVEYDWFLSE-------VKTALCLKDWIEEKDEDEICAKYGIAPGDLRRIVETAEWLSNAMNRIAEEVGNT------SVSGLTERIKHGVKEELLEL 634
Hel_Mva KINDMDRSNIDQ-YIFYIISKATEMRPLLRVK-----MDEELELIQE---MDKLKISDYS--IEN--IEAFKNSKMFLDWINETPEEVILEKYGIEPGIILRYKVDQARWMTYSSKEIAKLVLKLNNDKIYKALLEMEFRIEYGAKEELIEL 691
Hel_Sso EIA-----------YLHLLAFTPDGPLVSVGRN----EEEELIELLEDLECELLVEEPYEEDEYSLYLNALKVALIMKDWIDEVDEDTILGKYNIGSGDLRNIVETMDWLTYSAYHLSKELRLD--DHSDKLRILNLRVTDGVKEELLEL 652
Hel_Mka AVAS------------VRPFERFQLSPSIKRAVERVTRMSVPS-RLDDALSVIHSERKEVIERLPPKEKQKLVSLMKELDCGCDAFPHCEHVSQRASELALKVRLEGKSVYAIPRILEGRYGITAYPIDIANWLEEVVRL--LECAGEI 798
PolQ_Hsa LVTPMFEDWTT--IDWYRFFCLWEKLPTSMKRVAELVGVEEGFLARCVKG--KVVARTERQHRQMAIHKRFFTSLVLLDLISEVPLREINQKYGCNRGQIQSLQQSAAVYAGMITVFSNRLGWH--NMELLLSQFQKRLTFGIQRELCDL 930
PolQ_Mms LVTPVFEDWIS--IDWYRFFCLWEKLPTSMKRVAELVGVEEGFLARCVKG--KVVARTERQHRQMAIHKRFFTSLVLLDLISEIPLKDINQKYGCNRGQIQSLQQSAAVYAGMITVFSNRLGWH--NMELLLSQFQKRLTFGIQRELCDL 793
PolQ_Aor MFTPLQVAATTQ-VDWPIFRDQLDNLDESGLRALQFIGVQPGFVNRMVQSGASLKAECDTPEQIKLAVVYRRAYTALQLRDLSNEVPLSVVASRYRTPRGAVQTLAQQCHGFAAGMVKFCQRMGWG--MLAAVLDHMRDRLEAGARADLLEM 873
PolQ_Aae LVTPYSVSYQWQNISWVDFLERWENMDAAMRRVGELIGIRDAFLVKGLRG-----KIGEADHESLMTHKRFYTALALKRLVDEEPLSVVAQQFQCSRGLLQSLQQVASTFAGIVTAFCTSLNWN--LLAMIITQFRERLFFGVQPDLLDL 1137
PolQ_Tbr FVTPLRDPIDVD---WSIYRDIILSQLNETRQRIASMIGVDEFYVNQRAMG---LGDAGGNGGGRAFATRRFYAALALADLLNEVPIGEVEQRYRCNRGQLQNLLRSASMFSSITSFCRAMEWY--SLEAVLASFVKRLGFGVKPDIIPL 969
PIP-box
Hel_Pfu MQLPLVGRRRARALYNSGFRSIEDISQARPEELLKIEGIG--VKTVEAIFKFLGKNV-------KISEKPRKSTLDYFLKS 720
Hel_Afu VRIRHIGRVRARKLYNAGIRNAEDIVRHREKVASLIGRGI-AERVVEGISVKSLNPES 691
Hel_Mva LKIKNIGRVRARRLFNIGIKSKMDILKN-PE--KIISTFG--DKVGKKILDEFG-------------LKYGQQKLSGF--- 751
Hel_Sso VQIGGVGRKRARLLYNNGIKGLGDVVMN-PD--RVRNLLG--QKLGERVVQEAA-------------------RLLNRFH-- 708
Hel_Mka AEEPEMAAAAEALADPWSER- 818
PolQ_Hsa VRVSLLNAQRARVLYASGFHTVADLARANIVEVEVILKNAVPFKSARKAVDEEEEAVE(20)AAALIVEEARMILQQDLVE 1027
PolQ_Mms IRVSLLNAQRARFLYASGFLTVADLARADSAEVEVALKNSLPFKSARKAVDEEEEAAE(19)AAALIVEEAKMILQQDLIE 889
PolQ_Aor AQVTYVKGWTARLLRDNGFRNLRALAEADAKDLVPILMMVNPRKAQRSQTHTSEAERY(6)-KAEVIVASANKIWAFGVQD 956
PolQ_Aae MRIASLNGQRARLLFNAGVTGLLELANSDPLKIEQILYNCMSFETEQQRKGEEEFETR(20)AAHLLVTEARQYIQLEMGV 1234
PolQ_Tbr MEIKGVQPGRARALWRAGFKDPAAVAACTPAELLKRVKEANPPDSKVVKYFSIR--------SAVVVLREANAWVQQNIRE 1042
